# Water, sanitation, and hygiene service inequalities and their associated factors among urban slums and rural communities in Eastern Ethiopia

**DOI:** 10.3389/fpubh.2024.1438748

**Published:** 2024-11-13

**Authors:** Getachew Kabew Mekonnen, Abdurauf Zako, Fitsum Weldegebreal, Assefa Desalew, Temam Beshir Raru, Ukash Umer, Kedir Urgesa

**Affiliations:** ^1^School of Medical Laboratory Sciences, College of Health and Medical Sciences, Haramaya University, Harar, Ethiopia; ^2^Amir Nur District Health Office, Harar, Ethiopia; ^3^School of Public Health, College of Health and Medical Sciences, Haramaya University, Harar, Ethiopia; ^4^Laboratory Bacteriology Research, Faculty of Medicine and Health Sciences, Ghent University, Ghent, Belgium; ^5^Department of Pediatrics and Child Health Nursing, School of Nursing, College of Health and Medical Sciences, Haramaya University, Harar, Ethiopia; ^6^Department of Obstetrics and Gynecology, Leiden University Medical Centre, Leiden University, Leiden, Netherlands

**Keywords:** Eastern Ethiopia, hygiene, inequality, rural, sanitation, urban slum, water services

## Abstract

**Background:**

Understanding the level of inadequate water, sanitation, and hygiene (WaSH) services in urban and rural settlements is crucial for prioritizing community interventions and resource allocation. However, there is a lack of evidence regarding discrepancies in WaSH services across rural and urban slum communities in Ethiopia.

**Objective:**

This study aims to assess inequalities in households’ WaSH services and their associated factors among urban slums and rural communities in Eastern Ethiopia from February to April 2024.

**Methods:**

A comparative community-based cross-sectional study design was conducted, with study participants selected through stratified random sampling using proportional-to-size allocation. Data were entered into Epi Data version 3.1 and then exported to STATA version 17 for analysis. Bi-variable and multivariable logistic regression was conducted, and associations were reported as odds ratios (ORs) with 95% confidence intervals (CI), using a *p-*value less than 0.05 as the significance threshold.

**Result:**

A total of 278 urban and 301 rural households participated in the study, with a response rate of 94%. Key indicators showed that WaSH services were significantly better in urban areas compared to rural households. For example, 98% (95% CI: 96, 99) of urban households had access to an improved water source compared to 76% (95% CI: 71, 80) of rural households. The proportion of households with improved latrines was 44% (95% CI: 38, 50) in urban areas and only 14% (95% CI: 11, 19) in rural areas. Handwashing practices at all five critical times were reported by 52% (95% CI: 46, 58) of urban household heads, compared to 22% (95% CI: 18, 27) of rural households. Additionally, occupation (such as being a farmer, *p* = 0.000) and water service satisfaction (*p* = 0.000) were significantly associated with these key WaSH outcomes.

**Conclusion:**

The study revealed that the WaSH services in urban slums are considerably better than in rural households. Socioeconomic factors significantly influence the existing disparities in WaSH services. Stakeholders should focus on providing targeted, strategic support to communities to address the challenges in WaSH service provision.

## Introduction

Access to water, sanitation, and hygiene (WaSH) services is crucial and directly linked to the health of individuals and communities (1). It is also known that inadequate WaSH services have significant economic, environmental, and social impacts (2). In 2020, 74% of the global population used safely managed drinking water services, with 60% in rural areas and 86% in urban areas (3). Similarly, 54% of the global population used safely managed sanitation services, 44% in rural areas, and 62% in urban areas. Furthermore, 71% of the global population had basic handwashing facilities with soap and water at home (3). Despite global efforts, WaSH services are still inadequate in sub-Saharan Africa (SSA) (4). Sustainable Development Goal 6 (SDG6) targets universal and equitable services of safe and affordable drinking water, adequate sanitation and hygiene, and ending open defecation by 2030 (5). Inequalities in WaSH services exist in nearly all nations, affecting urban versus rural areas, the rich versus the poor, and marginalized and vulnerable groups (6). Poor people, in particular, are not only vulnerable but also at high risk of disease. In addition, inadequate WaSH disproportionately impacts women and girls living in urban slums (7).

According to the 2022 JMP WaSH report, urban areas in Ethiopia had 81% coverage for safe water, 65% for improved sanitation, and 83% for basic hygiene services. In contrast, rural areas had significantly lower coverage, with 62% for safe drinking water, 46% for improved sanitation, and 65% for basic hygiene (8). Water supply and sanitation are critical, as they involve hygienically isolating human excreta from human contact and drinking water sources to prevent contamination (9). The World Health Organization (WHO) defines improved sanitation facilities as those that effectively separate human excreta from human contact (10). Unsafe water and inadequate sanitation are major causes of diseases such as diarrhea and hookworm infection, posing serious risks to human health (11). In Ethiopia, only 68.7% of households have access to improved drinking water sources, 27.5% of them have improved toilet facilities, and only approximately 38% have handwashing facilities (12). Moreover, there is considerable variation in access to improved WaSH services across different regions of the country (13).

Multiple studies revealed that households with better economic status and education and urban households are more likely to use basic WaSH facilities (14). While such disparities have received significant attention, there are gaps related to other inequalities, such as gender, ethnicity, displacement, migration, and caste, which have been less measured and monitored (15). Many regions worldwide do not have proper facilities and funding to build or maintain WaSH infrastructure, especially in rural areas (16). However, inadequate WaSH services are more common in slum areas, areas dominated by informal settlements that are characterized by one or more of the five characteristics of overcrowding, poor sanitation, insecure land tenure, lack of access to water supply, poor housing quality and other infrastructure (17, 18). Reducing inequalities in all their forms is one of the key principles of the Sustainable Development Goal (SDG) and the principle of equality and non-discrimination, which applies to all human rights. However, there is a lack of evidence addressing the inequalities in access to WaSH services between the poorest rural society and urban slum communities in the study area. Therefore, this study aimed to evaluate inequalities in access to WaSH services and associated factors in rural and urban slum communities in Eastern Ethiopia.

## Methods and materials

### Study setting

This study was conducted in the Babile District of the Oromia Regional State and the Amir Nur District of the Harari Regional State. The two districts were randomly selected to represent the rural and urban slum communities. Harari Regional State is one of the regions in Ethiopia, located 526 km away from Addis Ababa to the east. This regional state includes nine districts. Amir Nur district is one of the urban districts with slum settlements in the Harari regional state that includes three urban slums, Kebeles, namely 01, 02, and 07, and it has 24,215 total population. The populations of 01, 02 and 07 Kebeles are 10, 674, 7,713, and 5,828, respectively. The urban Kebele 01 has an urban slum, Ganda Fero village and it includes 1,178 households with 685 vulnerable children under five. The other rural study sites were Bisidimo and Ifadin Kebelles in Babile district, which is located 540 km away from Addis Ababa and 23 km from Harar town in the eastern direction. The number of children aged from 1 to 15 years of age in Bisidimo and Ifadin Kebeles is 3,582, according to the unpublished Babile district administration report, 2023.

### Study design and period

A community-based comparative cross-sectional study was conducted from February to April 2024 in the Babile District of Oromia Regional State and the Amir Nur District of Harari Regional State, Eastern Ethiopia.

### Population

All households in rural and urban slum kebeles in Babile District of Oromia Regional State and the Amir Nur District of Harari Regional State, respectively, were the source population. The study populations were all randomly selected household heads within the selected rural and urban slum kebeles who were available during the study period. All household heads who were volunteers and lived at least 6 months in the study sites were included in the study.

Households that were closed at the time of the visit and heads who were not volunteers or had not lived in the area for at least the previous 6 months were excluded from the study.

### Inclusion criteria

All household heads who were volunteers and lived at least 6 months in the study site were included.

### Exclusion criteria

Households that were closed at the time of the visit and heads who are not volunteers and have not lived for at least the previous 6 months were excluded from the study.

### Sample size determination and sampling technique

The sample size was determined using a household survey formula where the proportion of 11.4 and 25% sanitation coverage in slum and rural areas of Ethiopia, respectively (19), with the assumption of precision or degree of error 0.05, confidence interval 95%, and non-response rate assumed to be 10%. We doubled the sample size considering the stratified analysis based on the type of residences and samples distributed proportionally to the number of rural and urban households in the Babile and Amir Nur districts, respectively. Thus, the rural district = 320 households, the urban district = 296 households, and the total sample size was 616. The target households were included in the study using systematic random sampling techniques.

### Operational definitions

#### Basic hygiene

Households with a handwashing facility with soap and water available on-premises meet the criteria for a basic hygiene service (20). Handwashing with water and soap at five critical times: before eating, after the toilet, after cleaning the child’s bottom, before preparing food, and before feeding the child.

#### Hand-washing practices at critical times

Washing hands with soap and water after toilet visits, after cleansing the child’s defecated buttock, before cooking (preparing food), before eating, and before feeding the child.

#### Improved sanitation

Improved sanitation facilities are those designed to hygienically separate excreta from human contact (20). These include wet sanitation technologies, such as flush and pour flush toilets connected to sewers, septic tanks, or pit latrines, and dry sanitation technologies, such as dry pit latrines with slabs and composting toilets.

#### Improved water source

Improved drinking water sources are those that, by nature of their design and construction, have the potential to deliver safe water that is accessible and available when needed (21). This includes piped water, boreholes or tube wells, protected dug wells, protected springs, rainwater, and packaged or delivered water.

#### Practice

For the practice questions, each correct response was valued as one, while incorrect responses were marked as zero. All the scores were added, and the mean score was calculated. Respondents who had scores below the mean value were categorized as having poor practice, while those who scored above the mean value were categorized as having good practice.

#### Urban slum

Urban settlements dominated by households of individuals living under the same roof lacking one or more of the following conditions: • Sufficient living area •Access to improved water •Access to improved sanitation •Durability of housing •Security of tenure (22).

#### WaSH service levels

The proportion of households that have access to an improved water source, an improved latrine, or those house heads practicing handwashing with water and soap at five critical times.

### Data collection and management

The project members developed a semi-structured questionnaire and an observation checklist with input from JMP (23). Fluent local language speaker numerators attended a 2-day training on the methods and tools. The data were collected using ODKA, and the tools were pretested on 5% of household heads in the non-selected Kebeles 7 days before the actual data collection to ensure the quality of the data collection tool. A questionnaire-based interview was conducted among heads, primarily mothers of the selected households, because women house heads have better knowledge about the existing WASH services. Spot checks of household WaSH services were held by a supervisor and the research team. The collected data was checked daily for completeness and consistency. Double data entry was done and cross-checked to ensure consistency. The investigators and supervisors coordinated all over the data collection process and took immediate corrective actions accordingly.

### Data analysis

Epi Data version 3.1 was used for data entry, and the data were then exported to STATA version 17 for analysis. Descriptive statistics were calculated and presented in tables and figures. Household WaSH services were compared, and a Chi-square test at a 95% significant level was computed to assess differences in access to basic WaSH services between urban slums and rural communities. Bi-variable and multivariable logistic regression analysis was conducted to identify associations between independent variables and outcome variables.

A variance inflation factor (VIF) was used to assess multicollinearity. Variables with a *p*-value less than 0.25 were included in the bivariate analysis and subsequently used in multivariable analysis to control for all potential confounders. Odds ratios (ORs) were calculated to determine the strength of the association. A *p*-value of ≤0.05 with a 95% confidence interval (CI) was considered statistically significant.

### Ethical considerations

Ethical approval was obtained from the College of Health and Medical Sciences Institutional Health Research Ethics Review Committee (Ref. No. IHRERC/056/24). Informed, voluntary, written, and signed consent was provided by house heads. The study group (househeads) who participated in the study was informed in the local language (Amharic and Afanoromo) about the study procedure and duration, following which they obtained consent. Data collected from the study group were kept confidential, and households had the right to withdraw from the study at any time. Necessary permissions were obtained from the district health office of Babile in east Hararghe of the Oromia region and the Amir Nur district health office of Harari regional state.

## Results

### Sociodemographic characteristics

A total of 278 urban and 301 rural households participated in the study, with a response rate of 94%. The mean income in urban slum households was 5,457.527 Ethiopian Birrs (SD: 2,617.87), whereas it was 3,401.015 Ethiopian Birrs (SD: ±1,312.1) in rural households. In this study, monthly income was used as an economic status indicator for households. Although income analysis of households may be inadequate, it is a cost-effective measure, and using a good sample makes it possible to analyze inequalities in a specific area. Households that earn more than the mean monthly income (4,404 Et. Birr) were considered to have a good income. Nearly 93% (532) of the house heads were married, while 70% (408) had no formal education (Table 1).

**Table 1:** Sociodemographic characteristics of households participated in WaSH inequalities assessment in Eastern Ethiopia, 2024 (*n* = 579).

Characteristic	Urban slum		Rural		Total
	Frequency	%	Frequency	%	Frequency
Village					
Ganda fero	139	50			139
Zerotwo	139	50			139
Bisidimo			151	50.2	151
Ifadin			150	49.8	150
House head age categories					
15 to 29 years	120	43.2	115	38	235
30 to 44 years	124	43.6	168	56	292
45 years and above	34	12.2	18	6	52
Household family size					
< 5	187	67	129	43	316
≥ 5	91	33	172	57	263
Marital status					
Single	0	0	1	0.4	1
Married	252	90.6	284	94.3	536
Separated/divorced	15	5.4	10	3.3	25
Widowed	11	4	6	1	17
Education					
No formal education	173	62	235	78.1	408
Primary school	44	16	25	8.3	69
Secondary school	7	2.5	26	8.6	33
College and above	54	19.5	15	5	69
Occupation					
Farmer	3	1.1	146	48.5	149
Merchant	86	3.1	15	5	101
Employed	87	3.1	28	9.2	115
Jobless	57	20.5	12	4	69
Housewife	39	14	96	32	135
Others	6	2.2	4	1.3	10
Household income per month					
Low income	95	34	237	78.7	332
Good income	183	66	64	21.3	247

### WaSH service inequalities

**Figure 1:**
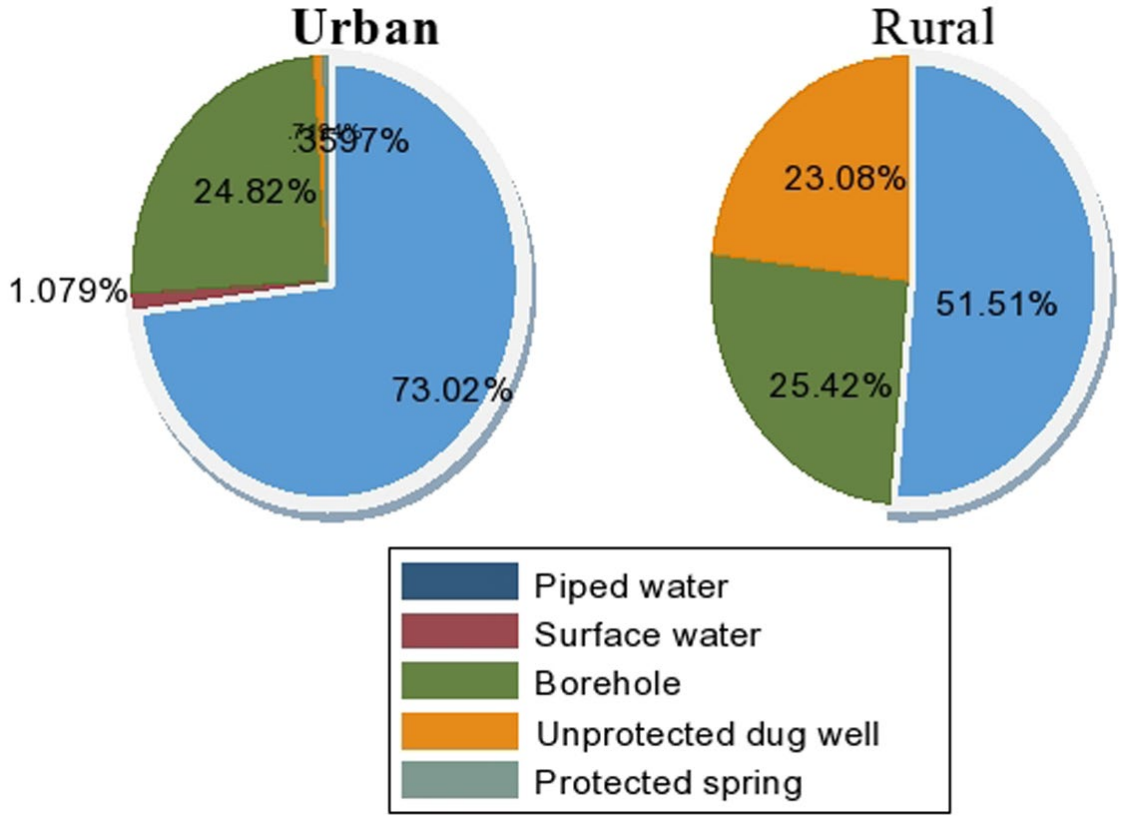
Type of drinking water sources in Amir Nur and Babile districts in Eastern Ethiopia, 2024 (*n* = 579).

The chi-square test showed that there are significant WaSH service inequalities in urban slums and rural households. More than 98% (95% CI: 96, 99) of the urban slum communities utilized improved water sources, while improved water source coverage was approximately 76% (95% CI: 71, 80) in rural households. The mean volume of water utilized per day *per capita* was 11.5 L. The mean distance of the household from the water source was 34.625 (SD: 112.6898) and 729.0317 (SD: 2458.032) in urban and rural areas, respectively. Water shortages were observed in 20% of urban and 48.7% of rural households within the previous month prior to the study. Approximately 8.3% of the urban households were satisfied with their water services, in contrast with 55% of the rural households (Figure 1). Nearly equal proportions, 90% of the urban and rural communities, did not get a standard minimum 20 L per day *per capita* water volume for drinking and domestic hygiene purposes. However, there was a greater trend of water service interruptions in rural areas than in urban areas.

**Figure 2:**
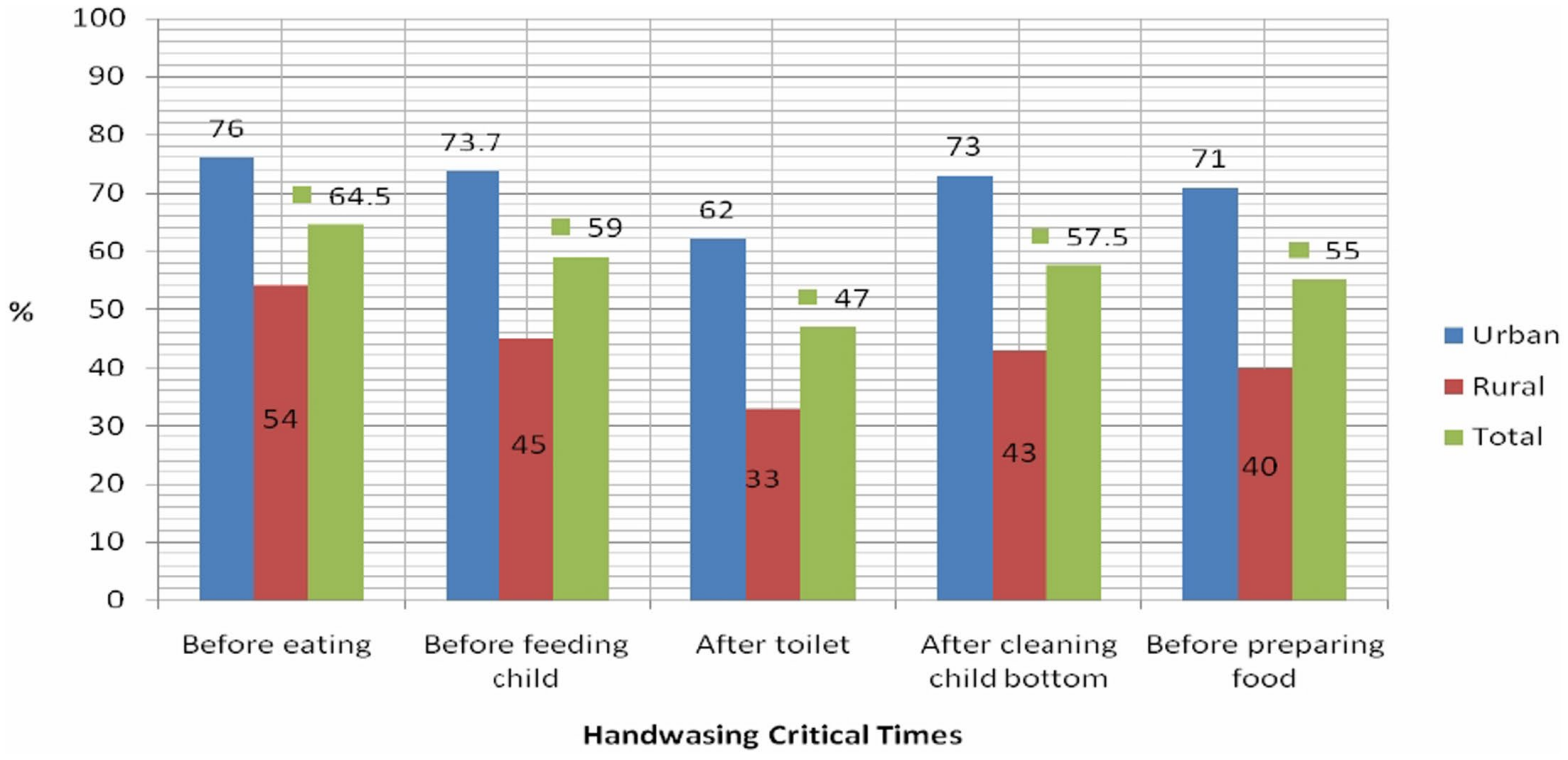
Hand washing practices of household heads at critical time in Amir Nur and Bisidimo districts in Eastern Ethiopia, 2024 (*n* = 579).

Approximately 81% of the urban slums and 76.6% of the rural households had some kind of toilet. Improved latrine was 44% (95% CI: 38, 50) in urban households and 14% (95% CI: 11, 19) in rural households. Furthermore, only 5.7 and 2% of urban and rural households had handwashing setups near their toilets, respectively. In contrast, the proportion of handwashing practices at all five critical times among urban house heads was 52% (95% CI: 46, 58), and it was 22% (95% CI: 18, 27) (Table 2). This is because the majority of the people in Eastern Ethiopia are Muslims and tend to frequently practice handwashing for religious reasons. However, they are mainly using portable small water containers instead of installing handwashing setups attached to the toilet. Similarly, the current study found that the most common handwashing practice was observed before eating food in both urban slums (71%) and rural (64%) communities (Figure 2).

**Table 2:** WaSH service inequalities among rural and urban slum communities in Babile District of Oromia Regional State and Amir Nur District of Harari Regional State, Eastern Ethiopia, 2024 (*n* = 579).

WaSH component	Urban (*n* = 278)		Rural (*n* = 301)		*X*^2^-test
	Frequency	%	Frequency	%	
Water access and adequacy indicators					
Source of drinking water					
Improved	273	98	230	76	0.000
Unimproved	5	2	71	24	
Type of water container used					
Narrow-mouthed	197	70.9	282	93.7	0.000
Wide-mouthed	13	4.7	8	2.6	
Both types	68	24.4	11	3.7	
The volume of water consumed per day *per capita*					
< 20 L	252	90.6	271	90	0.803
≥ 20 L	26	9.4	30	10	
Was there a water shortage within the previous month					
Yes	56	20.1	146	48.5	0.000
No	222	79.9	155	51.5	
Water source accessibility or distance					
< 500 m	277	99.6	239	79.4	0.000
≥ 500 m	1	0.4	62	20.6	
Households’ satisfaction with the water supply situation					
Satisfied	23		165	54.8	0.000
Not satisfied	255		136	43.2	
Sanitation indicators					
Availability of toilet facility for the household					
Yes	226	81	231	76.7	0.000
No	52	19	70	23.3	
Type of latrine used					
Improved pit latrine	122	43.9	43	14.3	0.000
Unimproved latrine	156	56.1	258	85.7	
Latrine distance from direct source of water					
≤ 30 m	211	93	30	13	0.000
>30 m	15	7	201	87	
Availability of handwashing setup					
Yes	15	5.7	6	2	0.029
No	263	94.3	295	98	
Availability of water in the handwashing setup					
Yes	14		5		0.481
No	1		1		
Availability of soap in the handwashing setup					
Yes	7		4		0.407
No	8		2		
Hygiene behavior indicators					
Were all water containers covered?					
Yes	132	47.5	108	35.9	0.000
Some only	140	50.3	184	61.1	
No	6	2.2	9	3	
Method of water drawing from storage					
Pouring	187	67.3	258	85.7	0.000
Dipping with cup	6	2.2	23	7.6	
Both pouring and dipping	72	25.9	18	6	
Spigot or tap	13	4.6	2	0.7	
When did you clean/empty the storage container before replacing it with fresh water?					
Today or yesterday	21	7.5	17	5.6	0.000
Less than a week	151	54.3	125	41.6	
A week ago	100	36	96	31.9	
Do not remember	6	2.2	63	20.9	
Were water containers clean?					
Yes	101	36	156	51.8	0.000
No	177	64	145	48.2	
Water treatment					
Yes	213	76.6	292	97	0.000
No	65	23.4	9	3	
Feces on the floor or walls of the latrine					
Yes	48	17.3	125	41.5	0.000
No	178	82.7	106	58.5	
Flies in the toilet					
Yes	115	41.4	211	70.1	0.000
No	111	58.6	19	29.9	
Feces on the ground in the compound					
Yes	174	62.6	142	47.2	0.000
No	104	37.4	159	52.8	
Routine handwashing critical times					
Before eating food	211	75.9	163	54	0.000
Before feeding children	205	73.7	135	44.8	
After toilet	172	61.9	100	33.2	
After helping and cleaning, children defecate	204	73.4	129	42.5	
Before preparing food	197	70.9	121	40.2	
Where does your family bathe usually?					
Bathroom	10	3.4	3	1	0.000
Toilet	94	33.8	13	4.3	
Inside home	147	52.9	177	58.8	
River and other	27	9.7	108	35.9	
Where do you dispose of household rubbish/solid waste?					
Waste bin/garbage	176	63.3	88	29.2	0.000
Open ground	57	20.5	147	48.8	
Farm/Bush	45	16.2	66	21.9	
Livestock in the household					
Yes	26	9.4	228	75.7	0.000
No	252	90.6	73	24.3	
Monthly income					
Low income	95	34.2	237	78.7	0.000
Goo income	183	65.8	64	21.3	

### Associated factors of WaSH services

In this study, we used Key JAMP WaSH service indicators—availability of an improved water source, availability of an improved latrine, and handwashing practices at five critical times—to assess the major factors associated with existing WaSH services in urban slums and rural households (21, 24). Accordingly, variables such as residence, age of respondents, educational status, occupation, monthly income, family size, and satisfaction with the water situation showed statistically significant associations with the three key WaSH indicators. All variables with a *p*-value less than or equal to 0.25 were considered for multivariable analysis. In multivariable analysis, variables such as residence, age of respondents, educational status, occupation, family size, and satisfaction with the water situation remained statistically significant at a *p*-value of less than 0.05 (Table 3).

**Table 3:** Associated factors of WaSH services.

Variable	Water source		AOR (95% CI)	*p* value	Latrine		AOR (95% CI)	*p* value	Handwashing practices at critical times		AOR (95% CI)	*p* value
	Unimproved	Improved			Unimproved	Improved			No	Yes		
Residence												
Rural	71	230	1		258	43	1		232	67	**1**	
Urban	5	273	42.2(11.8, 0.4)	**0.000**	156	122	5.8(2.5, 13.3)	**0.000**	132	146	10.4 (4.8, 22.2)	**0.000**
House head age category												
15–29 years	36	199	1		184	51	1		142	92	1	
30 and above years	38	254	1.5 (0.6, 4.1)	0.384	230	114	2.2 (1.3, 3.6)	**0.002**	222	120	0.84 (0.54, 1.3)	0.433
Education												
No formal education					326	82	1		273	132	1	
Primary school					33	36	3.3 (1.8, 6.3)	**0.000**	48	21	0.46 (0.24, 0.9)	**0.016**
Secondary school and above					55	47	2.9 (1.7, 5.1)	**0.000**	43	59	2.3 (1.3, 4.0)	**0.004**
Occupation												
Housewife	65	70	1		124	11	1		102	31	1	
Farmer	5	144	56 (17.6, 179)	**0.000**	123	26	3.0 (1.25, 7.3)	**0.014**	100	49	4.1 (1.98, 8.4)	**0.000**
Other	6	289	9.6 (3.03, 30.6)	**0.000**	167	128	2.1 (1.0, 4.4)	**0.049**	162	132	0.98 (0.5,1.8)	0.959
Family size												
1–5	32	284	1		222	94	1		178	135	1	
≥ 6	44	219	0.51 (0.19, 1.4)	0.195	192	71	1.2 (0.7, 1.86)	0.503	186	77	0.8 (0.53,1.2)	0.339
Water service satisfaction												
Satisfied	1	187	1		135	53	1		121	66	1	
Not satisfied	75	315	0.006 (0.001, 0.05)	**0.000**	278	112	0.39 (0.2, 0.756)	**0.006**	243	146	0.5 (0.3, 0.9)	**0.032**
Availability of toilet facilities												
Yes	55	401	1.03(0.4, 2.6)	0.984					259	195	1	
No	21	101	1						105	17	0.2 (0.1, 0.4)	**0.000**
Availability of handwashing setup												
Yes	1	20	0.16 (0.02, 1.58)	0.117	9	12	2.4(0.9, 6.4)	0.075	15	6	1	
No	75	482	1		404	153	1		349	206	0.29 (0.1, 0 0.8)	**0.022**
Livestock in the household												
Yes	60	194	0.54(0.19,1.57)	0.262	221	33	0.44 (0.2, 0.81)	**0.009**	194	59	1	
No	16	308	1		192	132	1		170	153	0.7 (0.4, 1.3)	0.294

Bold *p* values indicate that the factors have statistical significant association with the the outcome variable.

Generally, WaSH services are significantly higher in urban than rural households. This included access to an improved water source (AOR; 42.2, 95% CI; 11.8, 150.4), improved latrine facilities (AOR; 5.8, 95% CI; 2.5, 13.3), and handwashing practices at five critical times (AOR; 10.4, 95% CI; 4.8, 22.2). Types of occupation such as farmers (*p = 0.000*) and other jobs (*p = 0.000*) were significantly associated with existing improved water services compared to housewives. Furthermore, household satisfaction with water services (*p = 0.000*) was strongly associated with the availability of improved water sources (Table 3).

In terms of improved latrine availability, significant associations were found with the household head’s age (30 years and above, *p* = 0.002) and education levels—primary (*p* = 0.000) and secondary and above (*p* = 0.000). Additionally, occupations such as farmers (*p = 0.014*), other jobs (*p = 0.049*), household satisfaction with the existing water service (*p = 0.006*), and the availability of livestock in the household (*p = 0.009*) were significantly associated with the type of latrine used by the households.

In this current study, handwashing practices were assessed by categorizing participants who washed their hands at all five critical times—after using the toilet, before eating, before food preparation, after handling babies’ excreta, and before feeding a child—as having good handwashing practices. Participants who failed to wash their hands at any of these critical times were categorized as having poor handwashing practices. Significant associations were found with primary school education (*p* = 0.016), secondary school education (*p* = 0.004), occupation as farmers (*p* = 0.000), satisfaction with current water services (*p* = 0.032), availability of toilet facilities (*p* = 0.000), and availability of handwashing facilities (*p* = 0.022), all of which were linked to better handwashing practices at the five critical times.

## Discussion

Relevant actors in the WaSH domain now acknowledge that various social and economic inequalities mediate access to WaSH services (14, 15, 25, 26). The current study assessed these inequalities and found that virtually all indicators demonstrated significantly higher levels of WaSH services in urban slums compared to rural households. The proportion of households with good monthly income was also significantly higher in urban slums than in rural settings (*p* = 0.000). Rural populations typically have lower incomes than their urban counterparts in developing countries, a gap that can be attributed to disparities in education, job experience, and occupational categories (27).

It is well established that a household’s wealth index and monthly income significantly influence access to WaSH services (28), with wealthier households being more likely to have access to improved WaSH services compared to the poorest households (29, 30). This rural–urban inequality presents a significant challenge for authorities, who are encouraged to pursue spatially optimized policy reforms rather than implementing uniform nationwide measures.

The current study found that coverage of improved water sources was 98% (95% CI; 95.7–99.2%) in urban slums and 76% (95% CI; 71.2–80.6%) in rural communities. The availability of improved water sources was significantly higher in urban slums than in rural communities (AOR; 42.2, 95% CI; 11.8–150.4). While approximately 90% of households in both communities accessed less than 20 L of water *per capita* per day, water shortages were more prevalent in rural households than in urban slums. These findings, though varying in degree, align with the WHO SDGs five-year report (23).

Access to improved water sources was strongly associated with occupations, such as farming (AOR; 56, 95% CI; 17.6, 179) and other jobs (AOR; 9.6, 95% CI, 3.03, 30.6) when compared to housewives. This finding indicates a lower level of WaSH service awareness among housewives in the study areas. Additionally, households whose heads were dissatisfied with the available water services were approximately 100% less likely to have access to an improved water source compared to those who were satisfied. Household satisfaction with existing water services has been shown to have a positive and significant impact on ‘the improvement of WaSH services and the willingness to pay for those services (31).

Regarding sanitation, households in urban slums were six times more likely to have improved latrines compared to rural households, a finding consistent with other studies (23, 32). This result is in agreement with other similar studies and highlights the ongoing challenge of WaSH service inequalities in developing countries (33, 34). Our study showed that household heads aged 30 years and above were twice as likely to use improved latrines compared to those under 30 years. This finding is consistent with other similar studies and could indicate that the majority of individuals in this age group are economically active and better able to afford improved sanitation facilities (35).

Additionally, household heads with primary education (AOR; 3.3; 95% CI: 1.8, 6.3) and secondary or higher education (AOR; 2.9, 95% CI; 1.7, 5.1) were more likely to use improved latrines compared to those without formal education. This result mirrors findings from other studies, which suggest that educated household heads are more likely to adopt improved latrine practices than those without education (35, 36). Education plays a critical role in enabling individuals to make informed health decisions and to wisely allocate resources toward building and using improved latrine facilities (37).

Similarly, individuals working as farmers or in other occupations were at least three and two times more likely, respectively, to have improved latrines compared to housewives. This finding contrasts with other studies, possibly due to sociocultural differences between study areas. Additionally, households where the head was dissatisfied with the existing water service were 61% less likely to have improved latrines than those who were satisfied. Furthermore, households with livestock were 56% less likely to use improved latrines compared to those without livestock. In developing countries, farmers with lower levels of education are more likely to own livestock and less likely to build improved latrines, often resorting to open defecation (38).

In our study, good handwashing practices were defined as washing hands with water and soap at five critical times: before eating food, before preparing food, before feeding children, after using the toilet, and after helping or cleaning children. The results showed that good handwashing practices were 10 times more common in urban slums than in rural households (AOR; 10, 95% CI: 4.8, 22.2). This finding is consistent with other studies from Ethiopia (32). Households led by individuals with secondary and higher education demonstrated 2.3 times better handwashing practices than those led by individuals with no formal education, which aligns with findings from similar studies (39). These results are supported by other research (40, 41), likely because educated household heads tend to have greater awareness of the benefits of good hand hygiene practices.

Additionally, education fosters long-term changes in healthy behaviors and effectively promotes hygiene and sanitation (42). However, participants with only primary education were 54% less likely to wash their hands at critical times. This could be attributed to younger pupils in primary schools typically having incomplete information and a higher risk of poor personal hygiene (43). Surprisingly, farmers were four times more likely to practice good handwashing practices compared to housewives, possibly because housewives may perceive that their hands are clean as they usually stay home.

Household heads who were dissatisfied with the existing water service were 50% less likely to practice good handwashing at critical times, likely due to the necessity of household resources and adequate water services to maintain such practices (44). Households without toilet facilities were 80% less likely to have good hygiene practices compared to those with toilet facilities, as sanitation infrastructure promotes better hygiene practices among adults (45). Additionally, households lacking handwashing setups were 71% less likely to practice handwashing at critical times than those with available setups. This finding aligns with other studies (46), which suggest that the availability of handwashing setups significantly increases the likelihood of proper handwashing (41). To address these disparities, it is crucial to explicitly focus on reducing inequalities and targeting the poorest and most marginalized populations, as well as underdeveloped areas, in hygiene and sanitation efforts.

### Limitation of the study

This community-based study provided valuable insights into WaSH inequalities between urban and rural communities. However, certain key elements, such as the wealth index, were not assessed due to resource and time constraints.

## Conclusion

By examining the differences in access to basic WaSH between urban slums and rural areas access using selected household-level indicators, this study confirmed that WaSH services are significantly higher in urban slums than in rural communities. The study also identified several socioeconomic factors, such as sex, age, education of household heads, type of occupation, and monthly income, as affecting access to improved WaSH services. National and local governments should prioritize addressing these disparities responsibly, ensuring efficient resource allocation with a focus on equal opportunity and shared growth. Despite the differences, the limited availability of WaSH services in both urban and rural communities requires urgent attention. Collaborative efforts are required to maximize these services at the highest possible level. Therefore, stakeholders should focus on key socioeconomic and behavioral factors to help communities achieve the 2030 global WaSH goals and mitigate the significant health issues related to inadequate WaSH services.

## Data Availability

The raw data supporting the conclusions of this article will be made available by the authors, without undue reservation.
